# Influence of Heart Rate in Non-linear HRV Indices as a Sampling Rate Effect Evaluated on Supine and Standing

**DOI:** 10.3389/fphys.2016.00501

**Published:** 2016-11-15

**Authors:** Juan Bolea, Esther Pueyo, Michele Orini, Raquel Bailón

**Affiliations:** ^1^Centro de Investigación Biomédica en Red Bioingeniería, Biomateriales y Nanomedicina Zaragoza, Spain; ^2^BSICoS Group, Aragón Institute of Engineering Research (I3A), ISS Aragón, Universidad de Zaragoza Zaragoza, Spain; ^3^Institute of Cardiovascular Science, University College London London, UK

**Keywords:** HRV, ANS, HR-correction, nonlinear, entropy, D_2_, sampling rate

## Abstract

The purpose of this study is to characterize and attenuate the influence of mean heart rate (HR) on nonlinear heart rate variability (HRV) indices (correlation dimension, sample, and approximate entropy) as a consequence of being the HR the intrinsic sampling rate of HRV signal. This influence can notably alter nonlinear HRV indices and lead to biased information regarding autonomic nervous system (ANS) modulation. First, a simulation study was carried out to characterize the dependence of nonlinear HRV indices on HR assuming similar ANS modulation. Second, two HR-correction approaches were proposed: one based on regression formulas and another one based on interpolating RR time series. Finally, standard and HR-corrected HRV indices were studied in a body position change database. The simulation study showed the HR-dependence of non-linear indices as a sampling rate effect, as well as the ability of the proposed HR-corrections to attenuate mean HR influence. Analysis in a body position changes database shows that correlation dimension was reduced around 21% in median values in standing with respect to supine position (*p* < 0.05), concomitant with a 28% increase in mean HR (*p* < 0.05). After HR-correction, correlation dimension decreased around 18% in standing with respect to supine position, being the decrease still significant. Sample and approximate entropy showed similar trends. HR-corrected nonlinear HRV indices could represent an improvement in their applicability as markers of ANS modulation when mean HR changes.

## Introduction

Heart rate (HR) variability (HRV) has been studied as a non-invasive technique to assess autonomic nervous system (ANS) regulation of the heart. Although, HRV analysis is still controversial (Karemaker, 2006), its content has been related to sympathetic and parasympathetic modulation by Task Force of the ESC-NASPE (1996) and Sassi et al. (2015).

During the last decades, HRV analysis has been extended including nonlinear indices based on chaos theory. These methodologies describe ANS in terms of regularity and complexity. Non-linear indices have been studied in a wide range of cardiovascular diseases revealing discriminant power for risk stratification (Maestri et al., 2007). Correlation dimension was used to stratify women who suffered hypotension during spinal anesthesia in cesarean section (Chamchad et al., 2004; Bolea et al., 2014a). Sample and approximate entropy were studied comparing control vs. children with congenital heart malformation due to effects of cyanotic and acyanotic defects (Aletti et al., 2012). Furthermore, the integration of linear and nonlinear HRV indices has been shown relevant to stratify cardiac risk patients (Voss et al., 2009) and to describe pathophysiological mechanisms in the cardiovascular and neural system control (Signorini et al., 2011). Some studies pointed out that HRV complexity changes as a result of sympathetic activation (Porta et al., 2007; Turianikova et al., 2011; Weippert et al., 2013).

However, the physiological interpretation of HRV as a marker of ANS activity may be blurred since several factors could affect how intrinsic pacemaker cells and ANS activity are expressed in HRV (Yaniv et al., 2014a). The nonlinear relationship between temporal and complexity HRV indices with respect to HR has been addressed emphasizing the importance of attenuating this effect (Zaza and Lombardi, 2001; Platisa and Gal, 2006; Monfredi et al., 2014; Yaniv et al., 2014b). Furthermore, different mathematical models have demonstrated a relationship between HRV amplitude and HR correcting it (Chiu et al., 2003; Meste et al., 2005; Bailón et al., 2011; Sacha, 2014; Billman et al., 2015). HR correction effect on HRV analysis was studied to predict risk mortality (Pradhapan et al., 2014). Non linear indices, such as correlation dimension, sample, and approximate entropy, are computed over linearly detrended and normalized series so this effect is already compensated for (Osaka et al., 1993; Pincus et al., 1993; Porta et al., 2007; Voss et al., 2009; Bolea et al., 2014a). Despite this normalization, HR may still influence nonlinear HRV indices due to the fact that HR is the intrinsic sampling rate of HRV signal. This implies that the amount of information captured during the same time interval depends on HR. The dependence of nonlinear indices on data length has already been reported (Havstad and Ehlers, 1989), and some studies have computed nonlinear HRV indices over interpolated RR time series at different sampling rates to increase index reliability (Theiler, 1990; Osaka et al., 1993; Hagerman et al., 1996; Radhakrishna et al., 2000; Javorka et al., 2002; Kim et al., 2005). Our hypothesis is that the influence of HR as sampling rate on nonlinear HRV indices is still noticeable even when the same data length is considered.

The goal of this study is to assess and attenuate the HR influence as sampling rate on nonlinear HRV indices in order to provide insight in their physiological interpretation as markers of ANS modulation. To assess the influence of HR on nonlinear HRV indices, a simulation study is conducted in which changes in ANS modulation are independent of changes on mean HR. Based on simulation results, two approaches are proposed to attenuate this mean HR influence. Finally, HR-corrected nonlinear HRV indices are computed over a body position changes database to study their performance under ANS elicitation.

## Materials

### Body position changes (BPC) database

This database was developed collaboratively at Harvard Medical School, Massachusetts Institute of Technology, and the Favaloro Foundation Medical School. The whole cohort of short-term recordings comes from two data collecting studies. Further details of this database can be found in Sobh et al. (1995).

#### First study

Thirteen male subjects of age 21.6 ± 4.4 years (Mean ± SD; range, 19–38 years) with no history of cardiopulmonary disease participated in a study carried out at Clinical Research Center at the Massachusetts Institute of Technology, USA.

#### Second study

It comprises groups of subjects of different ages. Only the young group was included in our work (9 subjects, 26.7 ± 4.7 years; range, 20–35 years).

Thus, from the whole database we selected 22 subjects. Two recordings per subject were acquired containing 7-min electrocardiographic (ECG) and respiration (RP) signals, sampled at 360 Hz. The protocol included postural changes. First, ECG and RP signals were recorded while subjects were in supine position. Then, subjects changed to standing position and after 5 min, to allow reaching hemodynamic equilibrium, ECG and RP signals were recorded in standing position. Subjects were asked to breathe following an irregular sequence of tones (Sobh et al., 1995).

### Fantasia database

Twenty young rigorously-screened healthy subjects underwent 120 min of supine resting while continuous ECG and RP signals were recorded at 250 Hz while watching the movie Fantasia, Disney 1940, to help maintain wakefulness. Further database information is available elsewhere (Iyengar et al., 1996) and can be downloaded from http://www.physionet.org (Goldberger et al., 2000).

## Methods

### ECG preprocessing

Because the reliability of the HRV analysis can be compromised by low sampling frequency of ECG recordings (Merri et al., 1990), the ECGs belonging to BPC and Fantasia database were interpolated by cubic splines to a frequency of 1080 and 1000 Hz, respectively. Then, heartbeat times, *t(k)* where *k* symbolizes the *k*^*th*^ beat, were estimated using an ECG wavelet-based detector (Martínez et al., 2004). Ectopic beats were identified imposing a time-varying threshold on instantaneous heart rate variations. Then, these ectopic beats are corrected using the IPFM model, as described in Mateo et al. (Mateo and Laguna, 2003).

### Non-linear HRV analysis

Approximate, sample entropy and correlation dimension are methods that exploit the phase-space representation of a time series based on Taken's theorem (Takens, 1981). These nonlinear methods are described in the following and further mathematical details are provided in Appendix.

#### Approximate and sample entropy

*SampEn* and *ApEn* are irregularity measurements of the time series (Pincus, 1995). Although both entropies are closely related to each other, *SampEn* was introduced to overcome the self-pairs-related limitation of *ApEn* computation. Briefly, patterns of time series values (reconstructed vectors) of a certain length (embedded dimension, *m*) are compared to the rest of the possible pattern candidates. Those comparisons whose differences are below a threshold (*r*) are summed up and used to calculate correlation sums. The final entropy value measures the changes produced when increasing the length of the patterns in one unit. The parameters *m* and *r* have to be previously defined to estimate the entropy values. In this work parameter values are set to *m* = 2 and *r* = 0.15 for *SampEn*. For *ApEn, m* = 2 and *r* is set as the threshold that maximizes approximate entropy (*ApEn*_max_; Yentes et al., 2013). *ApEn*_max_ was selected instead of *ApEn* to avoid the bias introduced by in *ApEn* when considering self-comparisons (Mayer et al., 2016). Its computation is based on a previously published algorithm (Bolea et al., 2014a).

#### Correlation dimension

Correlation dimension, *D*_*2*_, measures the degree of complexity of the system that generates the time series (Grassberger and Procaccia, 1983). In a previous work, we developed techniques to improve the estimation of correlation dimension (Bolea et al., 2014a). On that study log-log curves (logarithm of correlation sums vs. logarithm of thresholds) were fitted to sigmoid curves, thus increasing the accuracy of maximum slope estimation. Moreover, another estimate of correlation dimension denoted as *D*_*2(max)*_ based on the points that maximize the difference between each pair of sigmoid curves was presented. Both *D*_*2*_ and *D*_*2(max)*_ were computed by varying *m* = 1–16 and *r* = 0.01–3 in steps of 0.01.

Non-linear indices estimation may be compromised when the amplitude value of time series appears discrete in a reduced set of values due to the lack of variation. A pre-processing stage is included and details can be found elsewhere (Bolea et al., 2014a).

### Simulation study

A simulation study is conducted to assess the mathematical relationship between HR and nonlinear HRV indices. The simulation study was carried out based on a HRV representation through the IPFM model. This model assumes that the ANS influence on the sinoatrial node can be represented by a modulating signal, 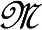
(t) (Mateo and Laguna, 2000). According to this model, when the integral of *1*+ 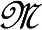
*(t)* reaches a threshold, *T*, a new heartbeat is generated at time instant *t(k)*. Threshold *T* represents the inverse mean HR.

Fantasia database was selected to compute modulating signals. Assuming that 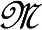
*(t)* is causal, band-limited and 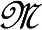
*(t)* < *1* then the instantaneous HR can be described as:

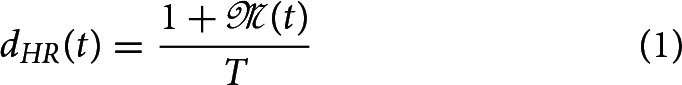



Instantaneous heart rate *d*_*HR*_*(t)* is obtained from the heartbeat times *t(k)* based on the IPFM model (Mateo and Laguna, 2003), and sampled at 4 Hz. A time-varying mean heart rate *d*_*HRM*_*(t)* is computed by low pass filtering *d*_*HR*_*(t)* with a cut-off frequency of 0.03 Hz. The heart rate variability signal is obtained as *d*_*HRV*_*(t)* = *d*_*HR*_*(t)* − *d*_*HRM*_*(t)*. Finally, the modulating signal, 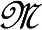
*(t)*, is approximated by $d_{HRV}\left(t\right)/\bar{d_{HRM}\left(t\right)}$ (Bailón et al., 2011), that is the HRV signal corrected or normalized by the mean HR.

Spectral analysis was applied to 5-min modulating signals 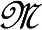
*(t)* by Welch periodogram. Frequency domain indices were estimated based on spectral bands (LF band from 0.04 to 0.15 Hz and HF band from 0.15 to 0.4 Hz). Respiratory frequency was checked to be within the HF band.

**Figure 1:**
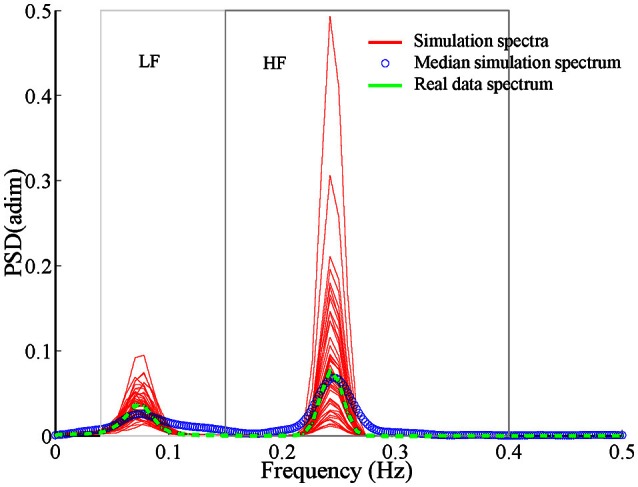
**Spectra derived from 50 stochastic realizations of simulated modulating signals during supine conditions (data simulates subject conditions from Fantasia database) applying ARMA technique fixing LF and HF content (red lines)**. Average spectrum is shown in circles (blue) and spectrum belonging to real data in dashed line (green).

Among all modulating signals, only those which presented one marked peak on each band (LF and HF band) were selected. Spectral indices such as the powers and the frequency peaks were used to generate synthetic modulating signals using an autoregressive moving average technique (ARMA; Orini et al., 2012). A total of one hundred 5-min segments were selected and their spectral indices were used to feed the ARMA model. A total of *M* = *50* stochastic modulating signals 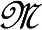 ^*j*^*(t)* with *j* = *1,…,M*, were simulated for each 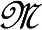
*(t)*. Figure 1 shows the spectra of 50 stochastic realizations, their median spectrum and the one of the segment-recording they are based on.

**Figure 2:**
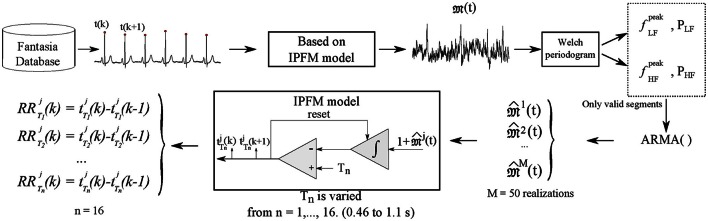
**Simulation data scheme is illustrated**. Heartbeat time occurrences are detected from ECG. Based on the IPFM model the ANS modulating signal 𝔐*(t)* is estimated. Spectral analysis by Welch periodogram is computed on 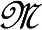
*(t)* in order to estimate the parameters needed for the simulation, frequency and power of LF and HF components, which are used to construct a new set of modulating signals, 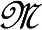 ^*j*^*(t)*, through ARMA technique, with *M* = 50 realizations. Then, IPFM model is used to generate simulated heartbeat occurrences $t{\;}_{Tn}^{j}\left(k\right)$ with different values of T from 0.46 to 1.1 s. Simulated RR series are computed from the $t{\;}_{Tn}^{j}\left(k\right)$.

Then, the IPFM model was applied on each stochastic realization, varying the parameter *T*_*n*_, where *n* = *1,…,16*, corresponding to T from 0.46 to 1.1 s in 0.04 s steps, to simulate the heartbeat occurrence times, $t{\;}_{Tn}^{j}\left(k\right)$, from which simulated 300-sample, are obtained. In this way, simulated RR series are generated where ANS modulation is independent from changes in mean HR. Simulation scheme is illustrated in Figure 2. *D*_*2*_, *SampEn* and *ApEn*_(*max*)_ are computed over these simulated RR time series.

Another simulation was done based on the BPC database characteristics. However, since subjects were asked to breathe following an irregular sequence of tones, the HF band does not show a dominant peak. In this case, the low and high frequencies used to feed the ARMA model were placed in the middle of LF and HF band, respectively. Then, modulating signals were simulated from spectral indices derived from supine and upright positions. This extends the analysis of HRV dependence on HR under enhanced sympathetic conditions.

### Non-linear indices dependence on HR as sampling rate

The methodology used to compute nonlinear HRV indices, considered in this study, is applied over linearly detrended and normalized RR time series. The detrending ensures that mean HR values are removed from the series whereas the normalization eliminates the influence of mean HR on HRV amplitude. Despite this fact, the effect of mean HR as sampling rate might still be present on them. In this section this effect is investigated on the simulation study, where changes in mean HR are independent from changes in ANS modulation. First, a mathematical relationship between nonlinear HRV indices and HRM is assessed by two regression formulas; then, a HR-correction is proposed based on these formulas. Second, interpolation of RR series is proposed to attenuate the sampling rate influence by mean HR on nonlinear HRV indices.

#### Regression formulas

In order to explore the relationship between nonlinear HRV indices and HR the following regression models were proposed.

(2) $$\begin{array}{r} \text{X}=\beta +\alpha RR,\left(\text{Linear model}\right), \end{array}$$

(3) $$\begin{array}{r} \text{X}=\beta \left(RR^{\alpha }\right),\left(\text{Parabolic model}\right), \end{array}$$

where X ∈ {*D*_*2*_, *SampEn, ApEn*}, α and β are regression coefficients.

Based on the former models HR-correction formulas were obtained by projecting each nonlinear index onto a standard level of *RR* = 0.5 s, hence:

(4) $$\begin{array}{r} \text{Linear}:{\text{X}}_{\text{C}1}=\text{X}+\xi \left(0.5-RR\right), \end{array}$$

(5) $$\begin{array}{r} \text{Parabolic}:{\text{X}}_{\text{C}2}=\text{X}{\left(\frac{0.5}{RR}\right)}^{\xi }, \end{array}$$

where ξ is the correction factor.

Transformation of X_*C*__1_ or X_*C*__2_ and RR into linear relationship was used to compute Pearson correlation coefficient ρ. Then, optimization was assessed by total least squares providing correction factors by the Golden Cut Search satisfying ρ(ξ) = 0.

Correction factors were computed on each stochastic realization. Thus, subject-specific correction was defined considering the correction factors of the 50 stochastic realizations for each modulating signal and computing the median of the HR-corrected indices.

Furthermore, a unique correction parameter was computed considering all stochastic realizations for all modulating signals. The transformation and optimization technique described above was applied to median values for each nonlinear index, thus defining a median correction approach to obtain *X*^ξ^_*C1*_ and *X*^ξ^_*C2*_.

#### Interpolation

RR series are unevenly sampled being the HR its sampling rate. This implies that the number of data information for the same time interval is dependent on HR. On the other hand, it is known that estimation of nonlinear indices such as correlation dimension, sample, and approximate entropy are dependent on data length (Havstad and Ehlers, 1989). Therefore, interpolating RR time series at the same sampling rate may alleviate the influence of mean HR on nonlinear HRV indices since it allows using the same number of data for the same time interval. Interpolation at 2, 4, and 8 Hz were studied (X_*I*2_, X_*I*4_, and X_*I*8_ respectively).

### Statistical analysis

Kolmogorov–Smirnov test was used to test the normality of data distributions. Mann–Whitney *U*-test was used when necessary; otherwise paired *T*-test was applied. Furthermore, Pearson correlation was used to assess linear correlation between corrected nonlinear HRV indices and *RR*. *p* < 0.05 are considered as statistically significant.

Bland–Altman plots were used to analyse the agreement of subject-specific vs. median correction formulas. The intra-classes coefficient (ICC) was computed by SPSS for Windows, Version 15.0. Chicago, SPSS Inc.

## Results

### Non-linear HRV indices and mean HR reflect body position-induced changes

**Figure 3:**
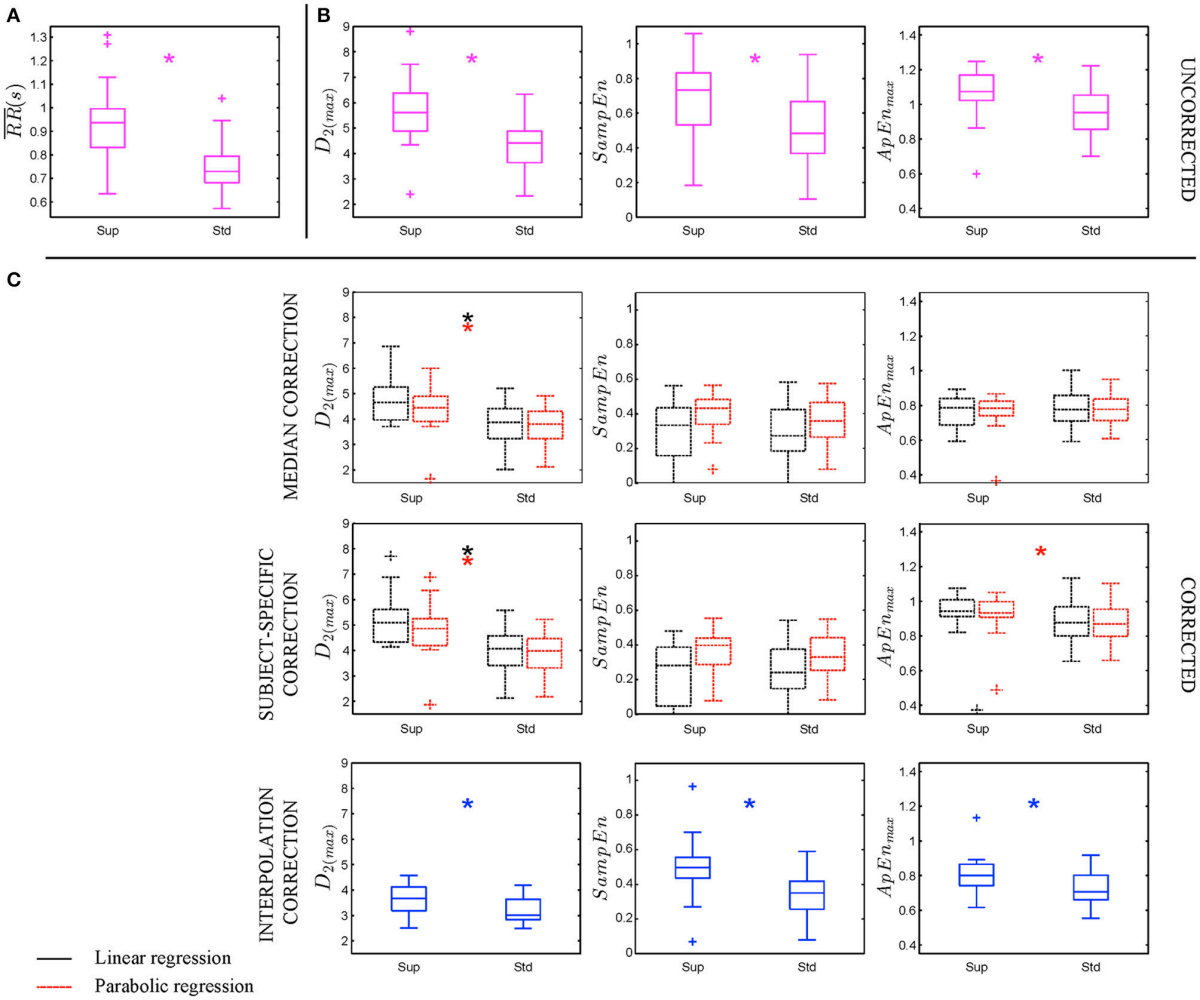
**BPC database (22 subjects) was analyzed in which supine and standing positions were compared. (A,B)** Illustrate mean RR and uncorrected nonlinear HRV indices while **(C)** shows HR-corrected nonlinear HRV indices. Asterisks (^*^) indicates *p* < 0.05 by Mann–Whitney *U*-test between supine (Sup) and standing (Std) positions.

Non-linear HRV indices (*D*_2_, *SampEn*, and *ApEn*_max_) were computed for the BPC database considering 300-sample segments. All of them were found significantly higher in supine than in standing position (see Figure 3B). Mean HR was also significantly higher in supine than in standing position (Figure 3A), which might explain the statistical differences observed in the computed nonlinear HRV indices.

### Relationship between non-linear HRV indices and RR in the simulation study

**Figure 4:**
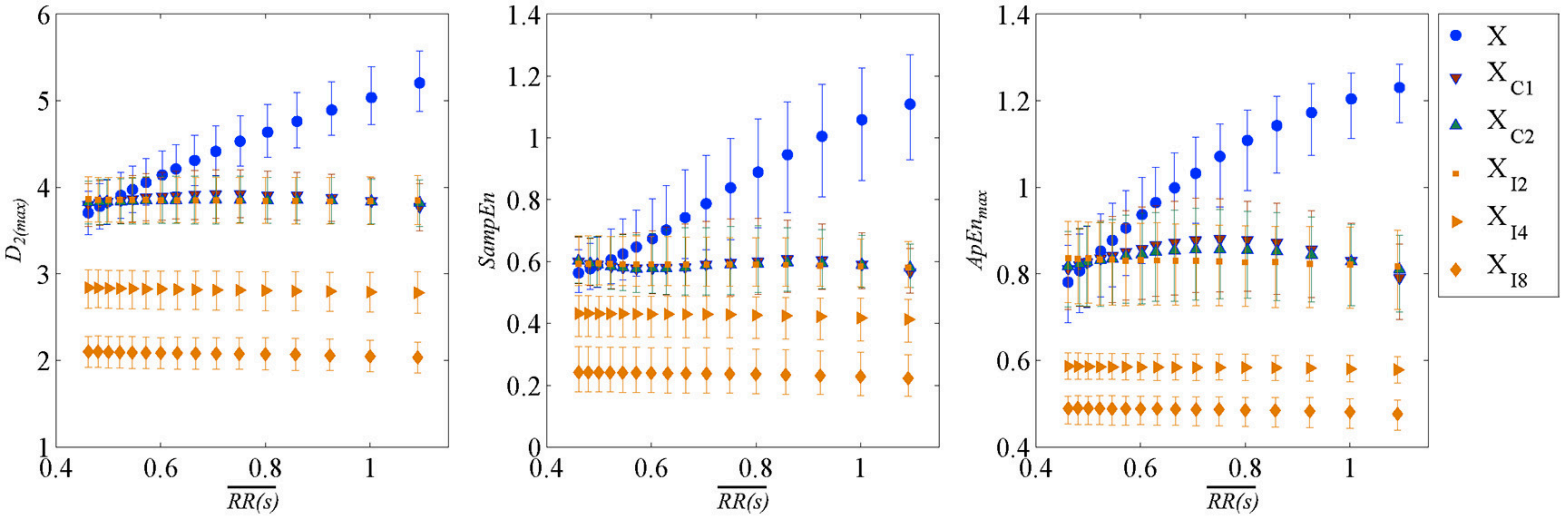
**Non-linear HRV indices computed from simulation study varying mean heart period, distribution of all uncorrected nonlinear HRV indices are shown in (blue) circles, corrected by linear regression in (brown) down triangles, corrected by parabolic regression in (green) up triangles, both former cases only subject-specific correction value of non-linear indices for each RR were shown**. Finally, HR-corrected non-linear indices by interpolating RR time series at 2, 4, and 8 Hz in (orange) diamonds. Data corresponds to all simulations derived from Fantasia database. Distributions are represented by median and interquartile range.

The relationship between nonlinear HRV indices and RR is assessed in the simulation study where RR is changed without changes in ANS modulation. Non-linear HRV indices computed from simulated data are illustrated in Figure 4 (median values shown as blue circles). The correlation of nonlinear indices and mean HR was evaluated by Pearson correlation coefficient finding high correlation, for a wide range of median index values being 3.5–5.02 for *D*_2(*max*)_, 0.42–1.02 for *SampEn*, and 0.72–1.24 for *ApEn*_max_ (see Table 1).

**Table 1:** **Pearson correlation factor ρ and ***p***-values of non-linear indices and RR obtained from simulation study**.

	**D**_**2(max)**_		**SampEn**		**ApEn**_**max**_	
ρ	0.959 ± 0.068		0.947 ± 0.1		0.949 ± 0.074	
*p*-value	0.0002 ± 0.0015		0.0004 ± 0.0044		0.0003 ± 0.0022	
Median ± IQR	4.26 ± 0.76		0.72 ± 0.30		0.98 ± 0.26	
**Regression formulas**	**Linear**			**Parabolic**		
	**D_2(max)C1_**	**SampEn_C1_**	**ApEn_C1_**	**D_2(max)C2_**	**SampEn_C2_**	**ApEn_C2_**
*R*^2^	0.919±0.129	0.896±0.186	0.902±0.139	0.923±0.129	0.896±0.179	0.910±0.125
ρ sub-spe (× 10^−05^)	0.013±0.20	0.016±0.21	−0.0051±0.20	−0.016±0.20	0.011±0.20	0.01±0.20
*p*-value _sub−spe_	1±0	1±0	1±0	1±0	1±0	1±0
Median ±*IQR*	3.88±0.07	0.59±0.02	0.85±0.04	3.85±0.02	0.59±0.01	0.84±0.03
$R_{\text{Median}}^{2}$	0.997	0.988	0.970	0.999	0.990	0.982
ρ_*Median*_ (× 10^−05^)	−0.787	−0.109	−0.051	0.661	0.232	0.061
*p*-value _Median_	1	1	1	1	1	1
ξ Correction factor	2.39	0.93	0.75	0.39	0.84	0.54
Median ± IQR	3.88±0.07	0.59±0.02	0.85±0.05	3.85±0.02	0.59±0.01	0.84±0.03
**2 Hz Interpolation**	**D_2(max)_**		**SampEn**		**ApEn_max_**	
ρ_*I*2_	−0.473 ± 1.41		−0.39 ± 1.09		−0.29 ± 0.77	
*p*-value *_*I*2_*	0.0005 ± 0.044		0.008 ± 0.19		0.068 ± 0.37	
Median ± IQR	3.85 ± 0.01		0.59 ± 0.002		0.83 ± 0.006	

*Linear and parabolic dependence of non-linear indices on RR was evaluated. Coefficient of determination (R^2^) as well as Pearson correlation factor and p-values (considering subject-specific and median correction) and ξ correction factor are presented for both regressions. Pearson correlation factor ρ and p-values of non-linear indices and RR obtained interpolating the simulated RR time series at 2 Hz. Data are given as median ± interquartile range*.

### HR-corrected non-linear indices by regression formulas

Regression formulas were applied to each simulated modulating signal (subject-specific approach) providing corrected indices with minimal mean HR correlation. The obtained HR-corrected nonlinear indices are shown in Figure 4 (median values considering all segments, in triangles right and left for linear and parabolic regressions, respectively). The application of correction formulas alleviated the correlation between nonlinear indices and mean HR. Furthermore, the range covered by them was highly reduced (3.81–3.95 for *D*_2(*max*)_, 0.57–0.61 for *SampEn*, and 0.8–0.9 for *ApEn*_*max*_), see Table 1.

**Figure 5:**
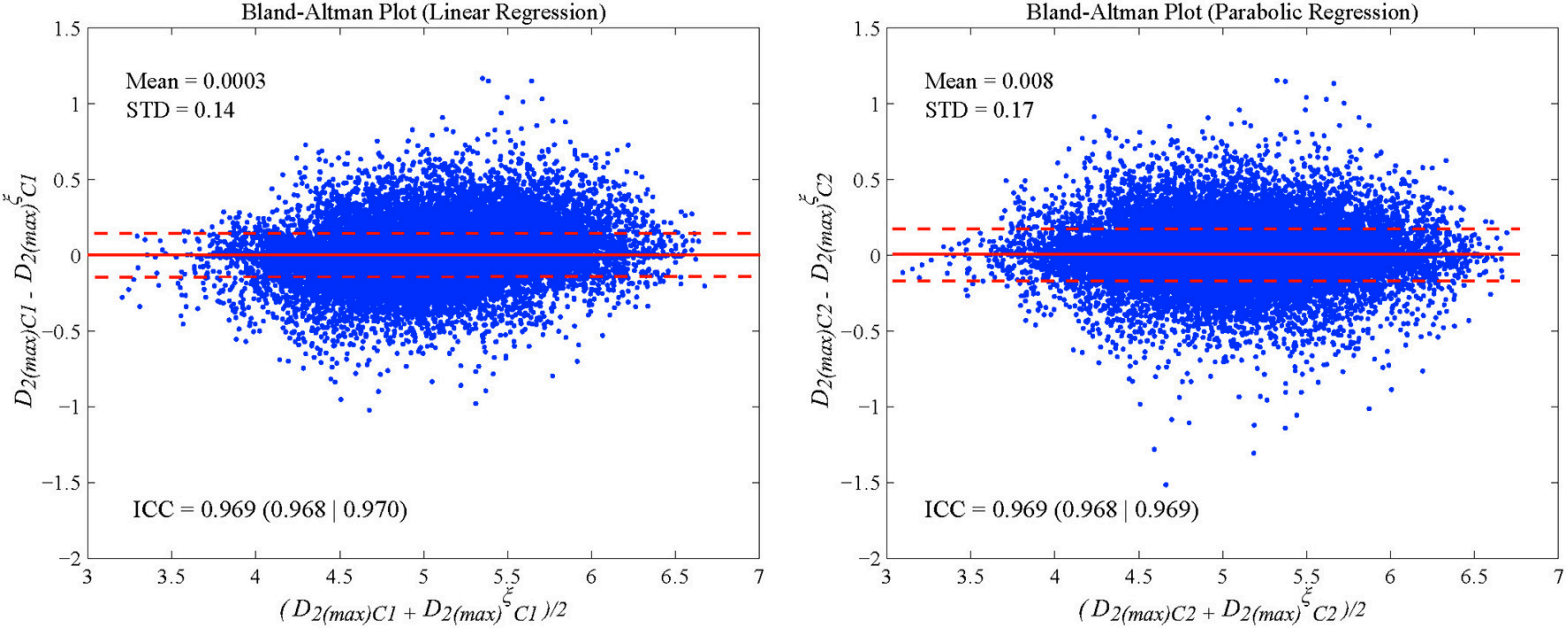
**Bland-Altman plots and intra-class coefficient (ICC) illustrate the agreement between subject-specific and median correction approaches computed on Fantasia database to correct HR effect for both proposed regressions for corrected ***D***_**2(***max***)**_ index: linear (left panel) and parabolic (right panel)**.

A set of correction factors (median approach) was obtained by considering the median of all nonlinear index values for each heart rate and computing global correction parameters (Table 1). To evaluate the agreement between subject-specific vs. median correction approaches, the ICC was analyzed, being above 0.8 for all HR-corrected nonlinear indices and for both proposed regression formulas. The Bland–Altman plot in Figure 5 illustrates the difference between both approaches in which *D*_2(*max*)_ is shown as an example.

### HR-corrected non-linear indices by interpolation

The nonlinear indices were computed from simulated RR time series resampled at 2, 4, and 8 Hz. As shown in Figure 4, the corrected nonlinear index values obtained applying regression formulas projected onto *RR* = 0.5 s and by interpolating RR time series at 2 Hz have similar median values. Despite the fact that Pearson correlation factor computed between HR-corrected nonlinear HRV indices by interpolation and mean HR is still significant their range is much reduced [3.84–3.86 for *D*_2(*max*)_, 0.588–0.592 for *SampEn*, and 0.824–0.836 for *ApEn*_*max*_] being negligible compared to the range of uncorrected nonlinear ones.

### Application to BPC database

The proposed HR-corrections were evaluated in the BPC database. The results shown in Figure 3C illustrate the differences found between supine and standing conditions. Median and interquartile range of uncorrected and HR-corrected nonlinear HRV indices are provided in Table 2.

**Table 2:** **Uncorrected Non-linear HRV indices and HR corrected by proposed approaches**.

	**Supine**	**Standing**	***p*-value**
*D*_2(*max*)*C*1_	5.61 (4.88|6.38)	4.41 (3.64|4.88)	0.0025
*D*_2(*max*)*C*1_	5.10 (4.33|5.62)	4.07 (3.41|4.57)	0.0014
*D*_2(*max*)*C*2_	4.85 (4.19|5.25)	3.97 (3.31|4.46)	0.0019
$D_{2{\left(max\right)}_{C1}^{\xi }}$	4.66 (3.98|5.27)	3.88 (3.24|4.42)	0.0045
$D_{2{\left(max\right)}_{C2}^{\xi }}$	4.47 (3.93|4.93)	3.82 (3.25|4.32)	0.0064
*D*_2(*max*)*I*2_	3.67 (3.23|4.08)	3.02 (2.84|3.64)	0.024
*SampEn*	0.73 (0.53|0.83)	0.48 (0.037|0.0.67)	0.008
*SampEn*_*C*1_	0.28 (0.05|0.38)	0.24 (0.15|0.37)	0.73
*SampEn*_*C*2_	0.40 (0.28|0.44)	0.33 (0.25|0.44)	0.44
$SampEn_{C1}^{\xi }$	0.333 (0.158|0.434)	0.272 (0.185|0.424)	0.39
$SampEn_{C2}^{\xi }$	0.436 (0.344|0.488)	0.364 (0.27|0.47)	0.062
*SampEn*_*I*2_	0.50 (0.42|0.54)	0.35 (0.26|0.42)	0.0013
*ApEn*_(*max*)*C*1_	1.11 (1.03|1.17)	0.88 (0.77|0.95)	0.008
*ApEn*_(*max*)*C*1_	0.94 (0.91|1.01)	0.88 (0.80|0.97)	0.057
*ApEn*_(*max*)*C*2_	0.94 (0.91|0.99)	0.87 (0.79|0.96)	0.038
$ApEn_{{\left(max\right)}_{C1}^{\xi }}$	0.784 (0.684|0.838)	0.775 (0.707|0.856)	0.5
$ApEn_{{\left(max\right)}_{C2}^{\xi }}$	0.783 (0.742|0.825)	0.777 (0.713|0.838)	0.94
*ApEn*_(*max*)*I*2_	0.80 (0.74|0.85)	0.71 (0.66|0.80)	0.0098

*Data is shown in terms of median and interquartile range. Statistical differences are tested by Mann–Whitney U-test*.

In a first study, the value of the median correction factor ξ extracted from the simulation study was used. It is worth noting that after linear correction there was no significant difference in *SampEn* and *ApEn*_*max*_ between supine and standing positions, while parabolic correction only reduced differences below significance for *SampEn*.

In a second study, simulation of each recording's characteristics was computed to apply subject-specific correction, derived independently from supine, and standing recordings. HR-corrected *D*_2(*max*)_ was found statistically significantly different for linear and parabolic regression formulas whereas *ApEn*_*max*_ was only significant for parabolic.

Finally, nonlinear HRV indices were computed on RR time series interpolated at 2, 4, and 8 Hz. We can conclude that the higher the interpolation order, the lower the nonlinear HRV values. In all cases HR-corrected nonlinear indices calculated by interpolation showed statistical differences between positions regardless of the interpolation order used (results of 4 and 8 Hz not included in the manuscript) being their range notably reduced.

## Discussion

HRV analysis has been widely used as a non-invasive technique to assess and quantify cardiac ANS modulation (Task Force of the ESC-NASPE, 1996; Voss et al., 2009; Sassi et al., 2015). However, HRV analysis is still under investigation due to HRV characteristics that could lead to physiological misinterpretations (Osaka et al., 1993; Chiu et al., 2003). ANS modulation is linked to ANS tone (HR mean) and, as a consequence, an increase in the sympathetic activity and a decrease in the vagal tone are related to an increment in the HR and a reduction on its variability (Chiu et al., 2003; Kazmi et al., 2016). This implies that there is a physiological correlation between HRV and HR. However, we have demonstrated that there exists also a methodological influence between nonlinear HRV indices and HR due to the fact that HR is the intrinsic sampling rate of HRV. To do this, we have conducted a simulation study. ANS modulating signals were generated as realizations of a stochastic process (Orini et al., 2012). Then, heart beat occurrences are generated using an IPFM model, which is based on action potential generation in SA node cells, and has been proven appropriate to describe the genesis of HRV (Mateo and Laguna, 2000). This simple model allows keeping the ANS modulation constant for different mean HR values, which is not possible in the reality. This simulation allowed assessing the nonlinear dependence of the standard deviation of normal beats on mean HR as it has been pointed out in previous studies (Zaza and Lombardi, 2001; Monfredi et al., 2014; Yaniv et al., 2014b; data not included in the manuscript). Two approaches have been proposed to attenuate the effect shown in the simulation study: regression formulas and interpolation.

### Regression formulas

Regression formulas are commonly used to characterize the relationship between two magnitudes such as ventricular repolarization and heart rate (Pueyo et al., 2004; Smetana et al., 2004; Baumert et al., 2010). The relationship between correlation dimension, sample, and approximate entropy, computed over simulated 300-sample RR series, and mean HR was studied by linear and parabolic regression. Although, the use of other regression models different from linear or parabolic ones may suppose an improvement, it would be unjustified since coefficients of determination *R*^*2*^ ≥ *0.9* were obtained for all cases for these models. Then, a correction was proposed based on regression formulas derived for each simulated case, the so-called subject-specific correction, minimizing nonlinear HRV indices correlation to mean HR. A correction based on regression formulas derived from median parameters was proposed as an extension to be applied to other databases. ICC values >0.8 were found when evaluating subject-specific vs. median correction approaches for all nonlinear HRV indices, suggesting the usage of either of the approaches, see Figure 5.

### Interpolation

Simulated RR time series were interpolated at 2, 4, and 8 Hz. The higher the interpolation rate, the lower the nonlinear index values. The addition of new data, resulting from interpolation, can be interpreted in terms of entropy as an increase in signal regularity being in concordance with a previous work in which electroencephalogram complexity through correlation dimension was evaluated varying the sampling rate (Jing and Takigawa, 2000). In our study, interpolation was used as a technique to alleviate the dependence of nonlinear HRV indices on mean HR as sampling rate effect since it allows estimating nonlinear indices over the same time interval and the same number of points. Sampling rate should be above the maximum HR. HR-correction nonlinear HRV indices computed by interpolating at 2 Hz and by regression formulas presented similar values and range. In some studies, RR time series were interpolated to increase the number of data points to increase reliability of nonlinear measurements, thus compensating mean HR effect on them. The used sampling frequency varies including 2, 4, and 8 Hz, or even 20 KHz (Osaka et al., 1993; Hagerman et al., 1996; Kim et al., 2005). However, since nonlinear HRV indices estimates are strongly dependent on the selected sampling rate, results should be compared with caution.

Despite the dependence of nonlinear HRV indices on mean HR revealed in the simulation study, no HR-correction of nonlinear HRV indices is applied in most of the studies found in the literature, where mean HR values are even not provided in some cases (Penttilä et al., 2003; Platisa and Gal, 2006; Melillo et al., 2011; Kunz et al., 2012; Moon et al., 2013; Weippert et al., 2013). The application of nonlinear indices without HR correction should be restricted to HR steady-state group conditions, as for example in Weippert et al. (2013).

Classical nonlinear HRV indices evaluated in the BPC database showed around 21, 34, and 21% of reduction in median values from standing with respect to supine position for *D*_2(*max*)_, *SampEn*, and *ApEn*_*max*_ respectively while mean HR increased by around 28%. Changes in these indices may reflect changes in mean HR as well as additional changes in ANS modulation, as suggested in a previous study (Platisa and Gal, 2006; Yaniv et al., 2014a).

In the BPC database, HR-corrected nonlinear indices were computed under supine and standing conditions and *D*_2(*max*)_ was found to be significantly different for all regression approaches while *ApEn*_*max*_ only for subject-specific using parabolic regression. Linear and parabolic regression formulas were selected to be suitable for the three indices under simulation conditions, although coefficients of determination were slightly lower for *ApEn*_*max*_ and *SampEn* than for *D*_2(*max*)_.

On the other hand, all nonlinear HRV indices were found still significantly different when corrected by interpolation. It was found a statistically significant reduction in standing with respect to supine position of 18, 30, and 12% for *D*_2(*max*)_, *SampEn*, and *ApEn*_*max*_ respectively, mostly reflecting ANS modulation changes while mean HR effect was attenuated. HR-corrected nonlinear index ranges, calculated as the difference of median values for supine and standing positions, were found reduced when compared to uncorrected nonlinear HRV index ranges.

Although, regression formulas were studied as HR-correction approach, their suitability depends on simulation requirements. Possible mismatches of simulated data with respect to real data difficult their application and therefore, we propose to compute nonlinear indices over interpolated RR series to attenuate mean HR effect, since no simulation is required, it saves computational time and still differentiates between both positions. This correction may lead to better understanding complexity and regularity under ANS changes unbiased by mean HR as natural sampling rate of RR time series.

Note that, although HR-correction attenuates the effect of mean HR as sampling rate, HR-corrected nonlinear HRV indices may be still correlated with mean HR due to their physiological dependence. After HR-correction, nonlinear HRV indices are capable of capturing information about ANS modulation in response to body position changes.

HR-corrected nonlinear HRV indices addressed in this study, pointed out a reduction in the complexity of the underlying system and an increase in the HRV series regularity caused by an increase of the sympathetic activity, when changing from supine to standing position, being in agreement with previous works with similar conditions, considering tilt table test or even exercise (Osaka et al., 1993; Kamen et al., 2000; Radhakrishna et al., 2000; Javorka et al., 2002; Porta et al., 2007; Bolea et al., 2014b). Nevertheless, these results and their physiological interpretation are limited by the low number of subjects of study and further studies are needed.

## Conclusion

In this work, changes in nonlinear HRV indices were studied under different sympathetic conditions where mean HR also changed. It is studied to what extend changes in nonlinear HRV indices are explained by HR ones. Correlation dimension, approximate and sample entropy dependence on mean HR as sampling rate is explored. A simulation study was carried out emulating ANS modulation no linked to mean HR. Simulation results showed that heart rate affects nonlinear indices as it is the intrinsic sampling rate of HRV even when considering the same data length. Two HR-correction methodologies, regression formulas and interpolation, were proposed. Their evaluation on a BPC database revealed a reduction of all studied HR-corrected nonlinear HRV indices in supine and standing positions. After HR-correction, nonlinear HRV indices are capturing changes in the sympathetic modulation by body position-induced changes. HR-correction by interpolation was found suitable to attenuate nonlinear HRV indices effect on mean HR and its application could represent an improvement in their applicability extending it in such cases of non-steady mean HR.

## Author contributions

All authors equally contributed to the conception of the work, revising it critically for important intellectual content, final approval of the version to be published, and agreement to be accountable for all aspects of the work in ensuring that questions related to the accuracy or integrity of any part of the work are appropriately investigated and resolved. EP and RB were supervisors of the research and MO gave methodological support. In addition, JB was responsible for drafting the work.

### Conflict of interest statement

The authors declare that the research was conducted in the absence of any commercial or financial relationships that could be construed as a potential conflict of interest.

## Appendix

### Non-linear method calculations

Let *x(k), k* = *1,…,N* be the time series of interest, *N* being the total number of samples. A set of *m*-dimensional vectors, *y*_*m*_*(i)*, called reconstructed vectors, can be generated:

(A1) $$\begin{array}{l} y_{i}^{m}={\left[x\left(i\right),x\left(i+1\right),x\left(i+2\right),\ldots ,x\left(i+\left(m-1\right)\right)\right]}^{T} \end{array}$$

Then, the amount of reconstructed vectors is *N*_*m*_ = *N* − *(m*−*1)* for each *m*-embedded dimension. The distance between each pair of reconstructed vectors, $y{\;}_{i}^{m}$, $y{\;}_{j}^{m}$ is denoted as $d_{i,j}^{m}$. The norm used is *L*_∞_. Then, each distance is compared with a threshold, *r*, in order to compute how many reconstructed vectors are within a hyper-space centered in the reconstructed vector of reference. The embedded dimension was set to *m* = *2* in this manuscript for the three following methods. Let see it mathematically on each particular case.

#### Approximate entropy (*ApEn*)

(A2) $$\begin{array}{l} C_{i}^{m}\left(r\right)=\frac{1}{N_{m}}\sum_{j=1}^{N_{m}}H\left(r-d_{i,j}^{m}\right) \end{array}$$

is the correlation sum where *H* is the Heaviside function.

(A3) $$\begin{array}{r} H\left(x\right)=\left\{\begin{array}{c} 1\;x\geq 0\\ 0\;x<0 \end{array}\right\} \end{array}$$

(A4) $$\begin{array}{r} \phi^{m}\left(r\right)=\frac{1}{N_{m}}\sum_{i=1}^{N_{m}}\log\left(C_{i}^{m}\left(r\right)\right) \end{array}$$

(A5) $$\begin{array}{r} ApEn\;\left(N,m,r\right)=\phi^{m}\left(r\right)-\phi^{m+1}\left(r\right) \end{array}$$

In addition, due to the intrinsic characteristics of *ApEn*, there is a threshold, *r*, for each time series at which *ApEn* is maximum defining *ApEn*_*max*_. Computation of *ApEn*_*max*_ is explained in the last subsection of this Appendix.

#### Sample entropy (*SampEn*)

Self-comparisons, $d_{i,i}^{m}$, bias *ApEn* estimation being more notable when data length becomes shorter. Thus, *SampEn* was introduced to solve this bias and to be data length independent. In this case,

(A6) $$\begin{array}{l} C_{i}^{m}\left(r\right)=\frac{1}{N_{m}-1}\sum_{j=1,j\neq i}^{N_{m}-1}H\left(r-d_{i,j}^{m}\right) \end{array}$$

is the correlation sum not considering self-comparisons.

(A7) $$\begin{array}{r} \phi^{m}\left(r\right)=\frac{1}{N_{m}}\sum_{i=1}^{N_{m}}C_{i}^{m}\left(r\right) \end{array}$$

(A8) $$SampEn(N,m,r)\;=\;-\text{In}(\phi^{m}(r)/\phi^{m\;+\;1}(r))$$

*SampEn* is also commonly calculated at the same range of *r* as for *ApEn*, spanning from 0.1 to 0.2 times standard deviation of the data.

#### Correlation dimension (*D2*)

(A9) $$C_{m}(r)=\frac{1}{N_{m}\left(N_{m}-1\right)}\sum_{i,j\;=\;1}^{N_{m}}H\left(r-d_{i,j}^{m}\right)$$

For deterministic systems, *C*_*m*_*(r)* decreases monotonically to 0 as *r* approaches 0, and it is expected that *C*_*m*_*(r)* is well approximated by $C_{m}\left(r\right)\approx r^{D_{2}^{m}}$. For correlation dimension estimation the threshold, *r*, is varied from *r* = 0.01–3 in steps of 0.01.

Thus, $D_{2}^{m}$ can be defined as:

(A10) $$\begin{array}{l} D_{2}^{m}=\underset{r\to 0}{\lim}\frac{\log C_{m}\left(r\right)}{\log\left(r\right)} \end{array}$$

For increasing *m*, $D_{2}^{m}$ values tend to saturate to a value *D*_2_ which constitutes the final correlation dimension estimate. Embedded dimension is varied from *m* = 1–16. In Bolea et al. (2014a), a fast computation algorithm was presented and it is further detailed elsewhere. The algorithm considers self-comparisons in the correlation sums allowing *ApEn* computation in a middle-step. Therefore, *ApEn*_*max*_ is computed at the same time as correlation dimension
