# Corrigendum: Influence of Heart Rate in Non-linear HRV Indices as a Sampling Rate Effect Evaluated on Supine and Standing

**DOI:** 10.3389/fphys.2018.00620

**Published:** 2018-05-28

**Authors:** Juan Bolea, Esther Pueyo, Michele Orini, Raquel Bailón

**Affiliations:** ^1^Centro de Investigación Biomédica en Red Bioingeniería Biomateriales y Nanomedicina, Zaragoza, Spain; ^2^BSICoS Group, Aragón Institute of Engineering Research (I3A), ISS Aragón Universidad de Zaragoza, Zaragoza, Spain; ^3^Institute of Cardiovascular Science University College London, London, United Kingdom

**Keywords:** HRV, ANS, HR-correction, nonlinear, entropy, D_2_, sampling rate

In the original article, we neglected to include the funder **(European Research Council**), **(ERC-2014-StG 638284)** to **(EP)**. The authors apologize for this error and state that this does not change the scientific conclusions of the article in any way.

The original article has been updated.

## Conflict of interest statement

The authors declare that the research was conducted in the absence of any commercial or financial relationships that could be construed as a potential conflict of interest.

